# A Case of Sarcoidosis with Interstitial Lung Disease Mimicking Clinically Amyopathic Dermatomyositis and Rapidly Progressive Interstitial Lung Disease

**DOI:** 10.1155/2014/195617

**Published:** 2014-11-09

**Authors:** Shinji Sato, Shinichi Nogi, Noriko Sasaki, Naofumi Chinen, Kiri Honda, Eiko Saito, Takayuki Wakabayashi, Chiho Yamada, Yasuo Suzuki

**Affiliations:** Division of Rheumatology, Department of Internal Medicine, Tokai University School of Medicine, 142 Shimokasuya, Isehara 259-1193, Japan

## Abstract

Here, we report a patient with sarcoidosis who developed edematous erythema and interstitial lung disease. At the initial visit, clinically amyopathic dermatomyositis (CADM) with rapidly progressive interstitial lung disease (RP-ILD) was suspected because he had progressive dyspnea but no muscle weakness. The presence of anti-CADM-140/MDA5 autoantibodies was immediately assessed to facilitate a precise diagnosis, with negative results. Thereafter, skin and transbronchial lung biopsies revealed noncaseating granuloma with Langhans giant cells in both specimens, leading to a diagnosis of sarcoidosis. In this case, clinical features of skin and lung were unable to distinguish DM (including CADM) from sarcoidosis, but the lack of anti-CADM-140/MDA5 antibody was useful for differentiating CADM with RP-ILD mimicking sarcoidosis from bona fide sarcoidosis.

## 1. Introduction

Sarcoidosis is a disorder of unknown etiology affecting multiple organs and is characterized by the formation of granulomatous lesions. It has many clinical manifestations including skin and pulmonary symptoms. In light of reported cases of sarcoidosis coexisting with dermatomyositis (DM) [1–6], it is important to distinguish between the two. Brateanu et al. reported a case of DM with diffuse micronodular infiltrations in both lungs and pathological findings showing sarcoid granulomatosis [3]. However, these cases seem to be rare. Here, we report a sarcoidosis patient who had skin erythema and acute onset interstitial lung disease (ILD) that was difficult to distinguish from DM and rapidly progressive lung disease (RP-ILD). In this case, testing for antibodies against CADM-140, also referred to as antimelanoma differentiation-associated gene 5 (MDA5) and known to be present in patients with DM and RP-ILD [7], was helpful in the differential diagnosis of sarcoidosis.

## 2. Case Report

**Figure 1:**
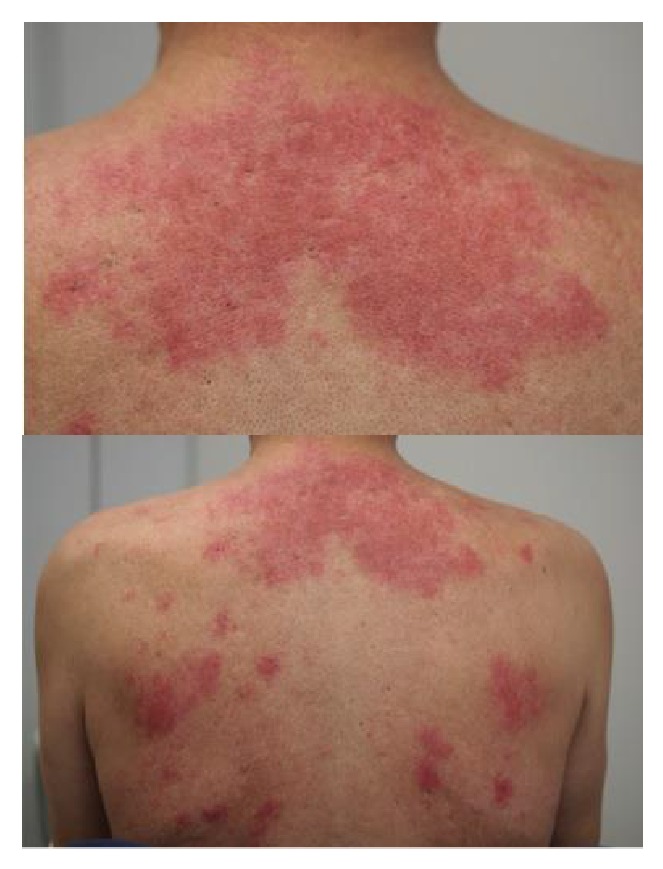
Clinical features of this case at the first visit. Diffuse indurated erythema was on his face, anterior chest, and dorsal region (figure).

**Figure 2:**
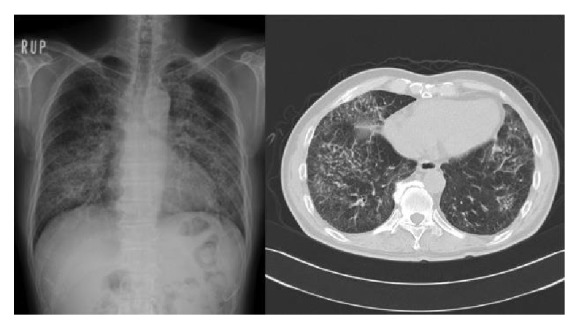
Chest radiography and CT findings at the first visit. Diffuse fine nodular or reticular shadow in both lung fields (predominant in lower lungs) was seen.

**Figure 3:**
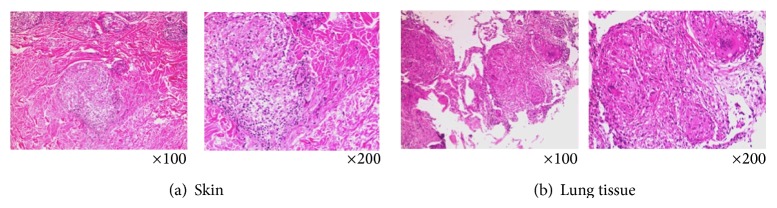
Pathological findings of the skin biopsy specimen from erythematous lesion of anterior chest (a) and TBLB specimen from lung (b) (hematoxylin-eosin staining, left: ×100, right: ×200). Infiltration of lymphocytes and epithelioid cells and noncaseating granuloma with Langhans giant cells were seen in both specimens. No caseous necrosis or malignant cells were seen.

A 63-year-old man had suffered from nonproductive cough and dyspnea on exertion since August 2010. In February 2011, he began to have a low-grade fever and he noticed erythema on his face, anterior chest, and dorsal region. Because his respiratory symptoms worsened rapidly, he consulted a general practitioner. Chest radiography and computed tomography (CT) revealed mediastinal lymphadenopathy, emphysematous change, and granular/nodular shadow on both lung fields. Blood chemistry revealed an elevation of serum LDH and serum KL-6. Because his respiratory symptoms were progressive, he was referred to our university hospital for further examination and treatment of the skin and respiratory symptoms. At the first visit, he had dyspnea on exertion and facial erythema as well as erythema on his anterior chest and back (Figure 1). He was afebrile, with no cervical, axillary, oringuinal lymph node swelling. Fine crackles were heard on both lung fields, although heart sounds were normal. No myalgia was present and no muscle weakness was detected in a manual muscle test. Laboratory findings revealed a white blood cell count of 6,600/*μ*L, red blood cell count of 517 × 10^6^/*μ*L, hemoglobin 17.0 g/dL, and platelets at 22.0 × 10^4^/*μ*L. Although serum AST was 56 IU/L, ALT was 57 IU/L, LDH was 260 IU/L, creatine kinase (CK) was 34 IU/L, and aldolase was 6.1 IU/mL, within thenormal range. Serum C-reactive proteins, KL-6 and SP-D, were elevated to 1.05 mg/dL, 1758 U/mL, and 279 ng/mL, respectively. Rheumatoid factor, antinuclear autoantibodies, and anti-Jo-1 antibodies were absent. Bacterial culture examination of sputum and thetuberculin skin test were negative. SpO_2_ (in room air) at rest was decreased to 92%. Chest radiography and CT indicated diffuse fine nodular or reticular shadow in both lung fields that suggested interstitial lung disease (ILD) (Figure 2). Electromyograms (EMG) indicated no myogenic changes, and a pulmonary function test revealed restrictive disorder. At that time, these clinical observations strongly suggested a clinical diagnosis of amyopathic DM (CADM), a subtype of DM which exhibits the DM rash without muscle weakness or myalgia, together with ILD (because the patient lacked typical DM-specific erythema such as Gottron's sign or Heliotrope rash and did not have any obvious muscle weakness). In order to exclude RP-ILD, we promptly tested for anti-CADM-140/MDA5 antibody, with negative results. Further examination showed that serum angiotensin-converting enzyme (ACE) was 51.8 IU/L (normal 7–25 IU/L) without elevation of serum tumor markers (CEA, ProGRP, and CYFRA). After testing for anti-CADM-140/MDA5 antibodies, a transbronchial lung biopsy (TBLB) was taken and bronchoalveolar lavage fluid (BALF) analysis was performed. BALF analysis indicated 3.28 × 10^5^ cells/mL with increased lymphocytes (49%) and an elevated CD4/CD8 ratio (8.40). TBLB and skin biopsy from the area of anterior chest erythema revealed infiltration of lymphocytes and epithelioid cells into the tissues and formation of noncaseating granuloma with Langhans giant cells. No caseous necrosis or malignant cells were seen. This is consistent with the pathological findings of sarcoidosis (Figure 3). Gallium scintigraphy indicated abnormal uptake into the right hilar lymph node and both lung fields. Together, these findings resulted in a diagnosis of sarcoidosis affecting the skin and lung. 30 mg of prednisolone daily was initiated and respiratory symptoms promptly improved in concert with the amelioration of skin manifestations. The patient remains well after gradually tapering the PSL dose without relapse of the disease or adverse events.

## 3. Discussion

This is a case report of sarcoidosis in a patient with lung and skin manifestations mimicking CADM and RP-ILD. Our case highlights the difficulty of diagnosing patients who manifest clinical features indistinguishable from DM (including CADM), particularly skin and pulmonary symptoms. Symptoms of both diseases sometimes closely resemble each other and it is difficult to distinguish them. Moreover, patients with both DM and sarcoidosis have been reported [1–6], although this seems rare. Most cases that are diagnosed as having both DM and sarcoidosis combine the pathological and clinical findings of both syndromes. However, our case was eventually diagnosed as having sarcoidosis solely on the basis of the pathological and imaging findings. Indeed, he had no typical DM skin rash and no pathological findings strongly indicative of DM. His clinical features could be explained by sarcoidosis.

Initially, this patient was suspected of having CADM and RP-ILD due to edematous erythema of the face and trunk and progressive dyspnea on exertion. RP-ILD accompanied by DM, especially CADM, is a life-threatening complication and it is well recognized that respiratory symptoms in these patients often progress rapidly and resist treatment even if intensive immunosuppressive therapy is given. However, it is also known that early intervention with high-dose corticosteroids and immunosuppressive agents can rescue patients from this life-threatening condition. On the other hand, sarcoidosis therapy usually does not require the type of intensive treatment that is necessary for DM and RP-ILD. In this regard, it would be important to differentiate sarcoidosis from DM in the early stages as quickly as possible. Anti-CADM-140/MDA5 antibody is a recently discovered myositis-specific autoantibody [7]. The presence of this type of autoantibody is now well known to be closely associated with DM and RP-ILD [7–9] especially in Eastern Asia. According to previous reports, frequencies of concomitant RP-ILD in patients with DM and anti-CADM-140/MDA5 antibodies range from 44 to 100% in Eastern Asia and from 18 to 57% in USA and Europe [10–23]. In our experience, 23 of 27 patients with DM and RP-ILD had anti-CADM-140/MDA5 antibodies (85%). Therefore, patients who possess anti-CADM-140/MDA5 antibodies have a high risk for having RP-ILD. The clinical usefulness of anti-CADM-140/MDA5 antibody for the evaluation of disease activity and the prediction of outcome has also been reported, as well as its utility for disease diagnosis [24]. The results for our case are consistent with the clinical significance of this antibody as well as confirming the utility of pathological examinations to differentiate DM and RP-ILD from other similar conditions such as sarcoidosis.

In summary, we report a sarcoidosis patient who developed edematous erythema and ILD mimicking CADM and RP-ILD. In this case, measurement of anti-CADM-140/MDA5 antibody was useful to distinguish sarcoidosis from CADM with RP-ILD. Successful treatment with PSL alone achieved complete remission of the sarcoidosis. This case emphasizes the importance of being aware of other conditions mimicking DM and RP-ILD, as well as the usefulness of measurement of anti-CADM-140/MDA5 antibody.
